# Hospital Culture and Healthcare Workers' Provision of Patient-Centered Care: A Moderated Mediation Analysis

**DOI:** 10.3389/fpubh.2022.919608

**Published:** 2022-06-06

**Authors:** Xianhong Huang, Yuan Gao, Hanlin Chen, Hao Zhang, Xiaoting Zhang

**Affiliations:** ^1^Department of Health Policy and Management, School of Public Health, Hangzhou Normal University, Hangzhou, China; ^2^Department of Administration, School of Public Administration, Hangzhou Normal University, Hangzhou, China

**Keywords:** hospital culture, healthcare worker, patient-centeredness, self-efficacy, achievement motivation

## Abstract

**Background:**

Patient-centered care (PCC) is globally recognized as a high-quality and high-value healthcare service. It emphasizes the broad participation of patients and families in health-related decision-making and the provision of healthcare services that cater to patients' needs, preferences, and values. However, the mechanisms driving healthcare workers' provision of PCC are yet to be fully uncovered.

**Methods:**

Using stratified random sampling, we recruited 1,612 healthcare workers from different levels of public hospitals in Hangzhou. We conducted survey interviews using questionnaires based on psychometrically sound scales. Structural equation modeling was used to analyze the effects of hospital culture, self-efficacy, and achievement motivation on the perceived provision of PCC by healthcare workers and to explore the mechanisms underlying their relationships.

**Results:**

Self-efficacy had a positive mediating effect in the relationship between hospital culture and healthcare workers' perceived provision of PCC (β = 0.424, *p* < 0.001). Furthermore, the pursuit of success positively moderated the mediating role of self-efficacy (β = 0.128, *p* < 0.001), whereas, the avoidance of failure negatively moderated the mediating role of self-efficacy (β = -0.017, *p* < 0.001).

**Conclusion:**

The findings suggest that hospitals should foster patient-centered and innovative cultures and develop strategies focusing on both internal motivation (self-efficacy and achievement motivation) and external environments (hospital culture) to help and encourage healthcare workers to implement PCC. For example, hospitals could further communication skills training, enhance leadership, build team spirit, and promote collaboration among healthcare workers.

## Introduction

The notion of patient-centered care (PCC) was first proposed by Balint in 1995 to express the belief that healthcare workers—who are involved in the process of treatment—should be familiar with patients' living conditions, social environments, and disease progression; that is, healthcare workers must deliver services that recognize and cater to the preferences, needs, and values of patients (1). PCC is a high-value healthcare service that is crucial to improving the quality of care and building harmonious doctor-patient relationships (2, 3). However, PCC calls for substantial competence among healthcare workers as it is complex and can be characterized as integrated medicine that is multi-leveled and comprehensive, covering the entire life cycle (4, 5). Therefore, clarifying the driving mechanism for the provision of healthcare is necessary to effectively intervene in the behaviors of healthcare workers. Furthermore, it is important to gain thorough and systematic insights into the factors driving PCC from different aspects, such as outer contexts and intrinsic motivation.

In recent years, many scholars have tried to elucidate the concept of PCC and explore the factors influencing behaviors of healthcare professionals pertaining to the provision of PCC; for example, in 2000, Ma studied the development and improvement of the hospital service system based on insights from the patient-centered approach, proposing a basic framework and solutions for the establishment of a patient-centered hospital service system (6). In 2002, Wang and Liu (7) argued that “patient-centeredness” involves improving doctor-patient communication, which can facilitate doctors' understanding of their patients and help doctors and patients reach a consensus on medical decisions. In 2019, Liang (8) summarized Western studies on patient-centered medical services and administration at the theoretical level and outlined practice guidelines for the implementation of PCC in China. Gender, grade, empathy, and communication skills were found to have statistically significant effects on dental students' attitudes regarding patient-centered services, which could be improved *via* a focus on enhancing empathy, emphasizing positive attitudes toward learning communication skills, and conducting patient-centered learning seminars (9). Furthermore, Kanat et al. (10) found that the doctor-patient relationship and communication, doctors' characteristics, and patients' engagement were important determinants of PCC. Paiva et al. (11) investigated the factors facilitating and inhibiting healthcare workers' implementation of PCC; they contended that the creation of an atmosphere that is conducive to communication, engagement of patients in medical decisions, and enhancement of medical personnel's ability to communicate effectively might foster the provision of PCC by medical workers.

Studies have linked PCC with various positive patient outcomes, including empowerment and engagement (10, 12) favorable health outcomes, diminished socioeconomic, and racial disparities, shorter hospitalization periods and earlier discharge, and lower treatment costs (13, 14). However, the existing literature on PCC has the following drawbacks. First, while many studies have identified various external (contextual) and internal (personal) factors associated with PCC provision, comparatively fewer studies have considered both contextual and personal factors holistically and identified the pathways linking them. It is extremely important to better understand how these factors interact to facilitate (or undermine) healthcare workers' provisions of PCC. Second, among the studies focusing on the factors influencing PCC, few have focused on organizational culture, which is an important factor that facilitates service ability by valuing people, stimulating new thoughts, fostering team spirit, and adopting systems that are recognized by employees. Third, studies focusing on the driving mechanisms of healthcare workers' provision of PCC have not investigated the impact of their intrinsic motivation. Thus, several studies so far have revealed that self-efficacy and achievement motivation have joint effects on personal behaviors (15, 16). However, few have been conducted in the field of hospital administration, and the mechanisms underlying the synergy between self-efficacy and achievement motivation are yet to be fully uncovered; for example, it is unclear whether hospital culture has different effects on the self-efficacy of and provision of care by healthcare professionals based on their level of achievement motivation. Therefore, the pathways of effects between hospital culture and healthcare workers' implementation of PCC are worth investigating.

Motives—both physiological and social—have been identified as major internal driving forces of human behavior in diverse domains (17). Achievement motivation refers to the perceived motivation that drives individuals to undertake challenging and meaningful work tasks and/or activities and surpass others to attain satisfactory outcomes (18). Thus, achievement motivation is an important internal variable that is positively associated with quality and initiative at work (19, 20). With regard to individual consciousness, achievement motivation is embodied in two opposing psychological tendencies: the pursuit of success and avoidance of failure (21). Schone (22) contended that strong achievement motivation is a positive predictor of employees' job engagement, work performance, and organizational behaviors, among others. Wang (20) explored the correlation between doctors' achievement motivation and sense of responsibility and found that achievement motivation is positively associated with service initiative. Feng (19) found that achievement motivation is one of the factors driving community practitioners to deliver first-contact services. Song et al. (23) showed that doctors with high achievement motivation maintain a positive outlook toward work, are friendlier with patients, and can overcome emotional exhaustion. According to McClelland's (24) theory of needs (also known as the theory of motivation), healthcare workers with a high (vs. low) level of achievement motivation are more devoted to work and are more focused, proactive, and persistent in delivering PCC owing to positive feedback (25).

In light of the aforementioned findings, we advocated the importance of considering the effects of achievement motivation while assessing the influence of hospital culture on patient-centered practice among medical staff. In addition, we included self-efficacy as an essential factor in the proposed model as this—as another intrinsic variable—could reflect healthcare personnel's level of confidence in the provision of PCC (26). Self-efficacy is an individual's belief in their capacity to set and achieve certain goals, that is, confidence in one's abilities (10). It is important to note that self-efficacy affects people's mindsets, responsiveness to emotions, and choice of behaviors (27). From a behavioral science perspective, self-efficacy might be a predictive factor for the adoption of and changes in health-related behaviors (28). For example, Ham and Tak (29) suggested that low self-efficacy could lead to avoidance behaviors and ineffective communication, resulting in poor clinical outcomes. Additionally, Afsar and Masood (30) found that enhancements in self-efficacy can facilitate innovative work-related behaviors such as PCC. Furthermore, Zhang et al. (31) demonstrated that self-efficacy is positively associated with health protection behaviors among pharmacists during the coronavirus disease 2019 pandemic.

Self-efficacy can be influenced by several contextual factors associated with hospital culture. For example, Meurling et al. (32) suggested that teamwork (or collaboration) and communication can contribute to the enhancement of self-efficacy among healthcare workers. Zhao et al. (33) proposed that the provision of external support to healthcare workers can enhance their self-efficacy and reduce job burnout. Additionally, the engagement and behaviors of leaders can influence the vitality, caliber, performance, and cohesion of their team (34). Xue et al. (35) found that leaders who favor and encourage changes can set a positive example for their coworkers and employees, fostering unity, and initiative in the organization; these factors are conducive to the organization's future. They also suggested that work atmosphere is positively correlated with initiative among employees. Furthermore, Liang and Gu (36) found that innovative organizational culture has positive effects on creativity and motivation (to perform) among employees. Therefore, an efficient hospital culture serves several functions—including guidance, bonding, stimulation, constraint, regulation, and security—which can contribute to the enhancement of self-efficacy among healthcare workers.

“Hospital culture” refers to the sum of collective consciousness, values, ethics, and norms in medical practice held by hospital employees under certain socioeconomic conditions (37). Studies have shown that hospital cultures based on internal communication, cross-team collaboration, innovation, and charismatic leadership (38–43) can facilitate the implementation of PCC by enhancing patient-centered consciousness among healthcare workers. Thus, the following hypothesis was formulated.

**Hypothesis 1 (H**_**1**_**):** Hospital culture has a positive effect on healthcare workers' implementation of PCC. In sum, on the one hand, hospital culture has a direct positive effect on the provision of PCC by healthcare professionals; on the other hand, self-efficacy mediates the effect of hospital culture on healthcare workers' implementation of PCC. Therefore, the following hypotheses were formulated.
**H**_**2**_: Hospital culture positively affects self-efficacy among healthcare workers.
**H**_**3**_: Self-efficacy among healthcare workers has a positive effect on the delivery of PCC.
**H**_**4**_: Self-efficacy mediates the relationship between hospital culture and healthcare workers' implementation of PCC.

Furthermore, achievement motivation moderates the relationship between hospital culture and healthcare workers' self-efficacy for the provision of PCC. Gong and Xue (44) explored the mechanism underlying the effect of empowering leadership on creativity among employees; they found that achievement motivation moderated the relationship between empowering leadership and self-efficacy. In addition, Sommaruga et al. (45) suggested that patient-centered practice among medical personnel can be enhanced by emotional intelligence. In sum, a good hospital culture has a relatively weaker positive effect on self-efficacy among healthcare workers with low achievement motivation. On the contrary, a good hospital culture has a stronger positive effect on the self-efficacy of healthcare professionals with high achievement motivation. Therefore, the following hypotheses were formulated.

**H**_**5**_: Achievement motivation moderates the relationship between hospital culture and self-efficacy among healthcare workers.
**H**_**6**_: Achievement motivation moderates the relationship between self-efficacy among healthcare workers and their implementation of PCC.

**Figure 1:**
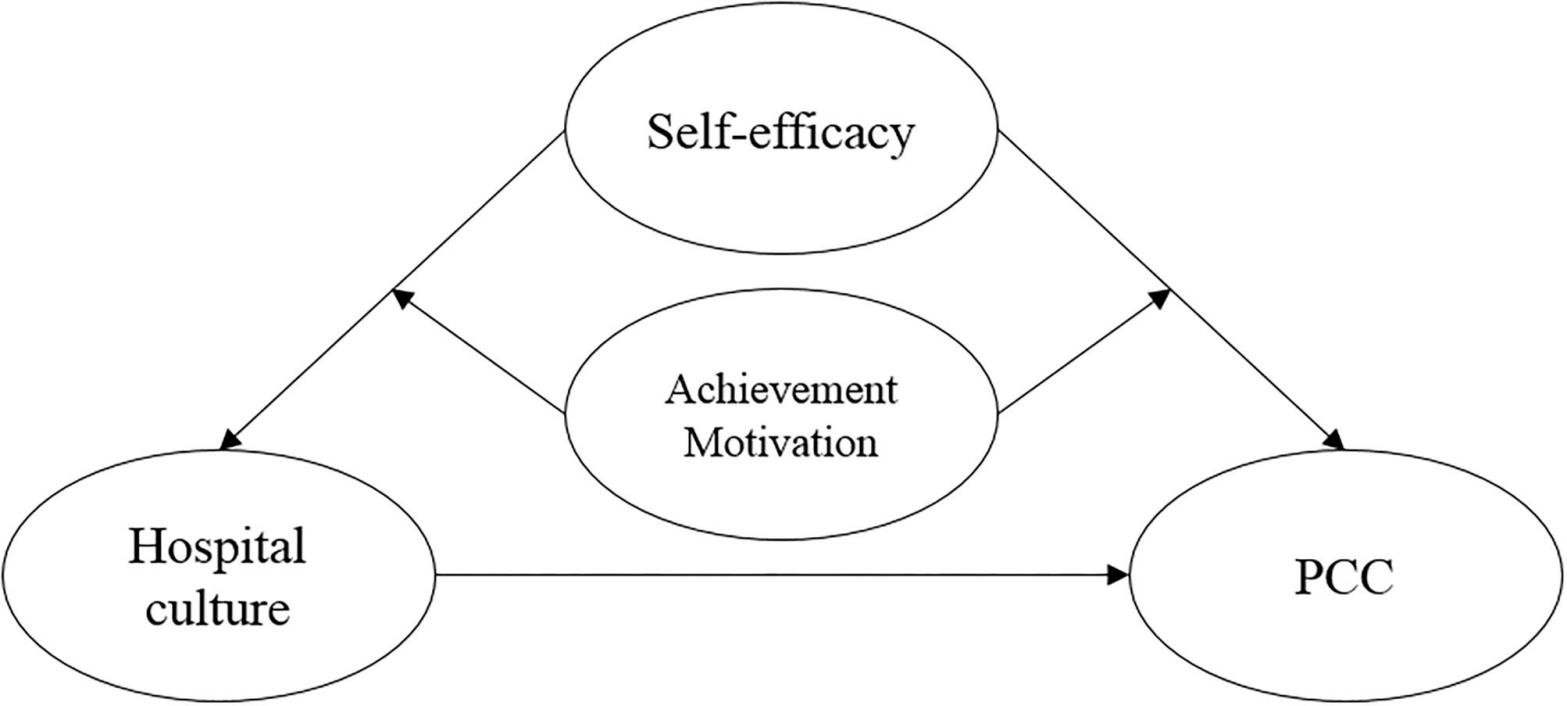
sResearch model diagram of healthcare workers' patient-centered care.

Thus, we surveyed healthcare workers from 27 public hospitals (of different levels) in Hangzhou to explore the pathways linking inner communication, cross-team collaboration, innovative organizational culture, charismatic leaders, self-efficacy, and achievement motivation to healthcare personnel's provision of PCC, considering intrinsic motivation and hospital culture. In light of theories of motivation, we proposed a theoretical model with hospital culture as the independent variable, healthcare professionals' implementation of PCC as the dependent variable, self-efficacy as the mediating variable, and achievement motivation as the moderating variable (Figure 1).

## Materials and Methods

### Sample Characteristics

Healthcare workers—including doctors, nurses, and medical technicians from different levels of public hospitals in Hangzhou city—voluntarily participated in this study. Hangzhou is a provincial capital located in coastal southeast China. It is economically well-developed and had a per capita GDP of 134,900 yuan in 2021. It is known for its quality of healthcare and high-ranked hospital administration, rendering it an apt setting for this study. The inclusion criteria were as follows: (1) providing informed consent; (2) being employed in the target hospital; and (3) having work experience of more than 6 months. The exclusion criteria were as follows: (1) being off-duty during the survey period owing to reasons like maternity leave, personal affairs, sick leave, learning, holiday, and/or business trips; and (2) questionnaires from interns or trainees from other organizations.

Stratified random sampling was used to select healthcare institutions based on three hospital levels; six tertiary and six secondary hospitals and 15 community health centers were randomly selected from among the medical organizations in Hangzhou. Convenience sampling was then used to select healthcare workers from each institution; 200 healthcare workers were selected from each tertiary hospital, 50 from each secondary hospital, and 20 from each community health center. Accordingly, 1,800 questionnaires were sent to these institutions through a face-to-face survey approach, of which 1,612 qualifying questionnaires were retrieved. The criteria for considering questionnaires invalid were: (1) incomplete responses; (2) same responses for more than 50% of the completed questions; and (3) obvious contradictions in responses across questions. If a questionnaire met even one of these criteria, it was deemed invalid.

### Measures

#### General Information

The form for general information comprised items pertaining to gender, marital status, age, level of education, title, post, department, years of experience, type of employment, daily working hours, monthly income, hospital level, teaching status of the hospital, communication skills training, and familiarity with PCC.

#### Provider-Patient Relationship Questionnaire

To evaluate the healthcare personnel's perceptions of their provision of PCC, we used the Provider-Patient Relationship Questionnaire (PPRQ), developed by Gremigni et al. (46). The PPRQ includes four dimensions: effective communication (four items), interest in the patient's agenda (four items), empathy (four items), and patient involvement in care (four items). Items are scored on a five-point Likert scale, ranging from one (never) to five (always); higher scores reflect better patient-centered services. The PPRQ has good reliability and validity (Table 1).

**Table 1:** Description, factor analysis, and reliability coefficients of construct measures.

**Construct**	**Dimension**	**Items**	**Load^a^**	**Cronbach's α**	**Correlation coefficient**	**Cumulative variance contribution rate (in %)**	**Overall α value**
Hospital culture	Internal communication	Group meetings are frequently organized in the department to discuss patients' conditions and treatment plans.	0.763	0.834	0.719^**^	75.201	0.944
		Resources and information can be shared within the hospital.	0.786		0.754^**^		
		Employees can freely point out when incidents with adverse consequences are likely to occur.	0.735		0.688^**^		
	Cross-team collaboration	The hospital encourages transdisciplinary cooperation.	0.862	0.902	0.791^**^	83.661	
		Members in the department have mutual understanding and acceptance.	0.86		0.783^**^		
		Different departments cooperate effectively and efficiently to solve patients' problems.	0.866		0.813^**^		
	Innovative organizational culture	Different departments in the hospital often collaborate with each other to develop innovative ways of providing health services.	0.822	0.936	0.830^**^	88.7	
		The hospital encourages constant innovation in health information technologies.	0.892		0.829^**^		
		The hospital encourages constant innovation in administrative skills and know-hows.	0.894		0.826^**^		
	Charismatic leadership	The leaders strive to set a good example for employees.	0.891	0.912	0.826^**^	85.148	
		The leaders emphasize that employees should show respect and concern toward patients and protect their rights.	0.845		0.833^**^		
		The leaders encourage employees to participate in discussions and decision-making processes.	0.851		0.802^**^		
Achievement motivation	Pursuit of success	I would be very happy to get recognized by patients.	0.859	0.92	0.588^**^	71.998	0.86
		I endeavor to provide personalized care for patients.	0.892		0.528^**^		
		I try to satisfy reasonable needs of patients and solve their problems.	0.915		0.538^**^		
		I prefer completing the work assigned to me as quickly as possible.	0.862		0.619^**^		
	Avoidance of failure	I feel uneasy when treatment, examination, and/or nursing don't show clear effectiveness.	0.821	0.851	0.586^**^	74.796	
		I would feel anxious if I am unable to reach a consensus with patients on decisions about treatment, examination, and/or nursing.	0.876		0.566^**^		
		I dislike dealing with incidents involving malpractices and/or disputes.	0.709		0.576^**^		
		I would feel anxious if I am unable to immediately understand questions asked by patients.	0.859		0.616^**^		
Self-efficacy	Confidence about interpersonal communication	I have a strong ability to build mutual trust with patients.	0.712	0.893	0.834^**^	69.321	0.942
		I have a strong ability to discern patients' emotions.	0.66		0.853^**^		
		I can solve patients' emotional issues and physical problems.	0.522		0.824^**^		
		Generally, I can be friendly with patients.	0.879		0.750^**^		
		I understand when patients do not approve of the treatment, examination, and/or nursing plan suggested by me.	0.643		0.745^**^		
	Confidence about effective communication	My patients would honestly tell me their medical history if I asked them.	0.586	0.911	0.834^**^	81.537	
		I have a strong ability to detect non-verbal hints or actions of patients.	0.816		0.853^**^		
		I possess the ability to ask pertinent questions at appropriate times.	0.802		0.824^**^		
		I can explain medical terms in simple language.	0.727		0.750^**^		
		Patients would be willing to communicate with me if I asked them about sensitive or private issues.	0.83		0.745^**^		
Patient-centered care	Patient engagement	In most cases, I provide detailed information regarding treatment, examination, and nursing to patients.	0.685	0.948	0.789^**^	78.435	0.967
		In most cases, I allot ample time for patients to consult with me.	0.72		0.780^**^		
		In most cases, I speak gently with patients.	0.558		0.818^**^		
	Empathy	In most cases, I patiently listen to patients.	0.632	0.933	0.866^**^	79.26	
		In most cases, I care about the extent of patients' understanding about their disease status and prognosis.	0.786		0.824^**^		
		In most cases, I inquire about patients' preferences and needs.	0.533		0.866^**^		
	Interest in patients' agendas	In most cases, I attach importance to the protection of patients' privacy.	0.802	0.905	0.795^**^	79.498	
		In most cases, I care about patients' expectations about the outcomes of care.	0.753		0.805^**^		
		In most cases, I can understand patients' negative emotions.	0.594		0.865^**^		
		In most cases, I can see things from the patients' perspective.	0.612		0.878^**^		
		In most cases, I can bring confidence and provide a sense of security to patients.	0.588		0.878^**^		
	Effective communication	In most cases, I can reach a consensus and resolve conflicts together with patients.	0.695	0.857	0.892^**^	83.36	
		In most cases, I encourage patients to get involved in discussions and decision-making about treatment, examination, and/or nursing.	0.78		0.825^**^		
		In most cases, I can resolve patients' concerns about their diseases and offer timely help.	0.774		0.877^**^		
		In most cases, I allow patients to ask questions and express their ideas when I ask about their symptoms.	0.764		0.876^**^		
		In most cases, I try to determine why a patient is reluctant to receive care.	0.766		0.821^**^		

a All load values are significant at the 0.001 level ^*^p < 0.05,

** p < 0.01, and ^***^p < 0.001.

#### Hospital Culture

A self-developed hospital culture questionnaire (38–43) was used to evaluate hospital culture based on four dimensions: internal communication (three items), cross-team collaboration (three items), innovative organizational culture (three items), and charismatic leadership (three items). Items are rated on a five-point Likert scale, ranging from one (strongly disagree) to five (strongly agree); higher scores represent better hospital culture. The scale had good reliability and validity (Table 1).

#### General Self-Efficacy Scale

Self-efficacy among healthcare workers was evaluated using the General Self-Efficacy Scale (GSES), developed by Schwarzer and Jerusalem (47). The GSES has two components: confidence about interpersonal communication (five items) and confidence about effective communication (five items). Items are rated on a five-point Likert scale, ranging from one (strongly disagree) to five (strongly agree), and higher scores represent higher self-efficacy. The scale had good reliability and validity (Table 1).

#### Achievement Motivation Measurement Scale

Healthcare workers' achievement motivation was evaluated using the Achievement Motivation Measurement Scale, developed by Huang (21). This scale includes two sub-dimensions: pursuit of success (four items) and avoidance of failure (four items). Items are rated on a five-point Likert scale, ranging from one (“strongly disagree”) to five (“strongly agree”); items in the avoidance of failure subscale are reverse scored. Higher scores reflect higher achievement motivation. The scale had good reliability and validity (Table 1).

### Quality Control

Prior to data collection, we conducted a pilot study. We gathered and analyzed the problems that were identified in the preliminary study. We then discussed the results of the preliminary study, revised the questionnaire, established a specific study plan, and finalized the questionnaire. Questionnaire distribution was performed by postgraduates with adequate experience in conducting personal surveys. The survey was conducted between July 1 and September 30, 2021. Furthermore, concentrated training sessions were conducted prior to the commencement of the official survey to ensure that the investigators had a clear understanding of the project, questionnaire, and key points during investigation; this process also ensured that they followed unified standards and methods. The survey was conducted *via* one-on-one interviews after obtaining informed consent from the participants. After the questionnaires were filled out, the investigators inspected them and checked with participants regarding the questionnaires that did not meet the study requirements. Subsequently, the questionnaires were coded and data were double-entered.

### Statistical Analyses

#### Preliminary Analyses

Data were coded and entered in the database. The demographic data were evaluated and compared by descriptive analysis, one-way analysis of variance, and independent *t*-test. In addition, SPSS version 22 (IBM Corp., Armonk, NY, United States) was used to test the Pearson's correlations between hospital culture, self-efficacy, achievement motivation, and PCC. Statistical significance was set at *p* < 0.01.

#### Mediation and Moderation Analyses

Structural equation modeling was used to analyze the mediating effect of self-efficacy among medical staff and the moderating effect of achievement motivation. Hayes' Process Macro Model 4 was employed to conduct mediation analysis, and Model 58 was employed for moderated mediation analysis (48). The aforementioned program is suitable for a variety of mediation, moderation, and moderated mediation models. Hypothesis testing for the regression coefficients was conducted using the bias-corrected percentile bootstrap method with 5,000 replicates at the 95% confidence interval (CI). If it did not include 0, the difference in effect was considered statistically significant.

### Ethical Considerations

The study was approved by the Institutional Review Board of Hangzhou Normal University. All participants provided informed consent, and the study was performed in accordance with the ethical standards as laid down in the 1964 Declaration of Helsinki and its later amendments.

## Results

### Demographic Characteristics

Of the 1,612 participants included in the study, 419 (54.3%) were male, 1,154 (71.6%) were married, and 182 (11.3%) were aged 46 or older. Additionally, 1,142 participants (70.8%) had a bachelor's degree, 602 (37.3%) had a junior title, 591 (36.5%) were doctors, and 501 (31.1%) were placed in the internal medicine unit. Moreover, 231 participants (14.3%) had work experience of 20 years or more, 1,199 (74.4%) held an officially budgeted post, and 1,175 (72.9%) worked for 8–10 h per day. Furthermore, 1,179 participants (73.1%) worked in tertiary hospitals and 1,259 (78.1%) in teaching hospitals; 581 participants (36.0%) had a monthly income of 5,001–7,000 yuan. Lastly, 958 participants (59.4%) had received training in patient-centered communication skills, 285 participants (17.7%) took 5–10 min on average to see one patient, and 132 participants (8.2%) were “very familiar” with PCC.

The healthcare workers' perceptions of their provision of PCC were evaluated based on their scores on the PPRQ (*M* = 66.70, standard deviation (SD) = 9.23). There were significant differences in participants' PPRQ scores based on age (*F* = 5.77, *p* < 0.01), title (*F* = 5.99, *p* < 0.01), post (*F* = 6.10, *p* < 0.01), and level of familiarity with PCC (*F* = 38.07, *p* < 0.01). Senior healthcare workers (M = 68.92, SD = 9.10) obtained higher scores than those with relatively junior titles (*M* = 67.18, *SD* = 8.88). Doctors (*M* = 67.98, *SD* = 8.55) obtained higher scores than nurses (*M* = 65.90, *SD* = 9.02). Furthermore, participants who were “very familiar” with PCC obtained the highest scores (*M* = 73.10, *SD* = 8.78), and those who were “very unfamiliar” with PCC obtained the lowest scores (*M* = 60.32, *SD* = 10.84) on the PPRQ. The PPRQ scores based on participants' demographic characteristics have been presented in Table 2.

**Table 2:** Comparison of healthcare workers' mean scores on the provider-patient relationship questionnaire based on different demographic variables.

**Variable**	**Categorization (*n*)**	**Total score**
Gender	Men (419)	66.74 ± 9.68
	Women (1,193)	66.68 ± 9.08
	*t* (*p*)	0.12 (0.90)
Marital status	Married (1,154)	66.97 ± 9.18
	Single (426)	66.05 ± 9.48
	Divorced (21)	66.24 ± 7.58
	Other (11)	64.09 ± 6.70
	F (*p*)	1.34 (0.26)
Age	≤ 25 years (229)	65.37 ± 10.12
	26–35 (712)	66.10 ± 9.09
	36–45 (489)	67.74 ± 8.83
	≥ 46 years (182)	67.91 ± 9.49
	F (*p*)	5.77 (<0.01)
Level of education	Junior college or below (221)	66.93 ± 9.10
	Graduate (1,142)	66.8 ± 10.64
	Postgraduate or above (249)	66.44 ± 9.10
	F (*p*)	1.37 (0.25)
Title	None (177)	66.01 ± 9.41
	Junior (602)	65.57 ± 9.46
	Middle (556)	67.18 ± 8.88
	Sub-senior (211)	68.53 ± 8.93
	Senior (66)	68.92 ± 9.10
	F (*p*)	5.99 (<0.01)
Department	Internal medicine (501)	66.20 ± 9.13
	Surgery (125)	67.35 ± 8.55
	Gynecology and obstetrics (96)	67.88 ± 7.37
	Pediatrics (64)	68.53 ± 8.56
	Medical technologies (230)	65.34 ± 11.25
	Emergency room (70)	65.50 ± 8.71
	Ophthalmology and otorhinolaryngology (27)	65.26 ± 10.14
	Psychiatry (105)	67.38 ± 8.32
	Other (394)	67.46 ± 8.93
	F (*p*)	2.24 (0.05)
Daily working hours	<8 (277)	65.99 ± 9.54
	8–10 (1,175)	66.73 ± 9.21
	10–12 (128)	67.30 ± 9.16
	> 12 (32)	69.31 ± 6.99
	F (*p*)	1.59 (0.19)
Post	Doctor (591)	67.98 ± 8.55
	Nurse (744)	65.90 ± 9.02
	Medical technician (256)	66.08 ± 10.75
	Other (21)	66.62 ± 11.88
	F (*p*)	6.10 (<0.01)
Level of hospital	Tertiary (1,179)	67.77 ± 9.87
	Secondary (204)	65.15 ± 10.34
	Community health center	66.86 ± 8.75
	F (*p*)	3.31 (0.05)
Teaching status of the hospital	Teaching hospital (1,259)	66.83 ± 9.19
	Not a teaching hospital (353)	66.23 ± 9.37
	*t* (*p*)	1.07 (0.28)
Level of familiarity with patient-centered care	Very unfamiliar (56)	60.32 ± 10.84
	Quite unfamiliar (132)	63.47 ± 10.17
	Fairly familiar (639)	64.42 ± 9.09
	Quite familiar (653)	67.97 ± 8.09
	Very familiar (132)	73.10 ± 8.78
	F (*p*)	38.07 (<0.01)
Work experience (years)	<1 (183)	66.19 ± 9.11
	1–5 (372)	65.97 ± 9.53
	5–10 (402)	66.35 ± 9.25
	10–15 (287)	66.78 ± 8.91
	15–20 (137)	68.21 ± 8.83
	> 20 (231)	67.88 ± 9.32
	F (*p*)	2.20 (0.05)

The means, SDs, and correlation coefficients of the variables under study have been presented in Table 3. Hospital culture was positively associated with self-efficacy (*r* = 0.549, *p* < 0.001) and healthcare workers' perceived provision of PCC (*r* = 0.662, *p* < 0.001). Similarly, there was a significant positive association between self-efficacy and healthcare workers' perceived provision of PCC (*r* = 0.559, *p* < 0.001). Additionally, significant positive correlations were observed between the variables (hospital culture, self-efficacy, pursuit of success, avoidance of failure, and PCC).

**Table 3:** Means, standard deviations, and correlation coefficients of the variables.

**Variable**	**Mean**	**SD**	**Hospital culture**	**Self-efficacy**	**Pursuit of success**	**Avoidance of failure**	**Patient-centered care**
Hospital culture	48.561	7.584	1				
Self-efficacy	38.891	6.228	0.549^***^	1			
Pursuit of success	16.899	2.647	0.630^***^	0.638^***^	1		
Avoidance of failure	9.627	2.989	−0.270^***^	−0.326^***^	−0.400^***^	1	
Patient-centered care	66.478	9.387	0.662^***^	0.559^***^	0.628^***^	−0.265^***^	1

^*^p < 0.05, ^**^p < 0.01, and ^***^p < 0.001. SD, standard deviation.

### Analysis of the Mediating Effect of Self-Efficacy

First, a mediation model was constructed with hospital culture as the independent variable and the perceived provision of PCC as the dependent variable after controlling for age, title, post, and level of familiarity with PCC (Table 4). The results of Equation 1 showed that hospital culture had a significant positive effect on healthcare workers' perceived provision of PCC (β = 0.815, *p* < 0.001). Bootstrapping was performed using 5,000 bootstrap replicates (as parameter estimation). The 95% CI was 0.772–0.859 and did not include 0; thus, the results supported H1, suggesting that hospital culture had a positive effect on healthcare workers' perceived provision of PCC.

**Table 4:** Testing the mediation model of self-efficacy.

**Variable**	**Equation 1 (PCC)**			**Equation 2 (Self-efficacy)**			**Equation 3 (PCC)**		
	**β**	**SE**	***t***	**β**	**SE**	***t***	**β**	**SE**	***t***
Constant	23.609	1.339	17.635^***^	13.877	0.983	14.113^***^	17.721	1.344	13.186^***^
Age	0.628	0.280	2.241^*^	0.200	0.206	0.974	0.543	0.266	2.038^*^
Title	0.765	0.248	2.813^*^	0.765	0.182	4.197^***^	0.373	0.237	1.576
Post	−0.993	0.229	−4.342^***^	−0.627	0.168	−3.733^***^	−0.727	0.218	−3.331^***^
Familiarity with PCC	0.520	0.191	2.730^*^	0.599	0.140	4.282^***^	0.266	0.182	1.461
Hospital culture	0.815	0.022	36.681^***^	0.446	0.016	14.113^***^	0.626	0.025	24.764^***^
Self-efficacy							0.424	0.031	13.612^***^
R^2^	0.466			0.345			0.517		
	F (5,1724) = 300.384^***^			F (5,1724) = 181.710^***^			F (6,1723) = 307.960^***^		

^*^p < 0.05, ^**^p < 0.01, and ^***^p < 0.001. PCC, patient-centered care.

Second, self-efficacy was entered into the model as the mediating variable. The results for the mediating effects of self-efficacy have been depicted in Table 4 (Equations 2, 3). Hospital culture had a significant positive effect on self-efficacy (β = 0.446, *p* < 0.001) with the 95% CI within the 0.414–0.478 range; thus, the results supported H2, validating that hospital culture positively influenced self-efficacy among healthcare workers. As shown in Equation 3, self-efficacy had a significant positive effect on healthcare workers' perceived provision of PCC (β = 0.424, *p* < 0.001); the estimated 95% CI was within the 0.363–0.485 range, and did not include zero. Thus, the results supported H3, reflecting that self-efficacy among healthcare workers had a positive effect on the delivery of PCC. Self-efficacy had a partial mediating effect in the relationship between hospital culture and PCC implementation. The size of the mediating effect has been presented in Table 5. Additionally, 76.81% of the total effects of hospital culture on the perceived provision of PCC were direct effects and 23.19% were indirect effects, mediated by self-efficacy. These results supported H4, suggesting that self-efficacy mediated the relationship between hospital culture and healthcare workers' PCC implementation.

**Table 5:** Total, direct, and mediating effects of self-efficacy in the relationship between hospital culture and healthcare workers' perceived provision of patient-centered care.

	**Effect size**	**Boot SE**	**Lower end of boot CI**	**Upper end of boot CI**	**Percentage**
Total effect	0.815	0.022	0.772	0.859	
Direct effect	0.626	0.025	0.577	0.676	76.81%
Mediating effect of self-efficacy	0.189	0.024	0.143	0.238	23.19%

*Boot, bias-corrected percentile bootstrap method; SE, standard error; CI, 95% confidence interval. All values have been rounded up to three decimal places*.

### Analysis of the Moderating Effect of Achievement Motivation

**Figure 2:**
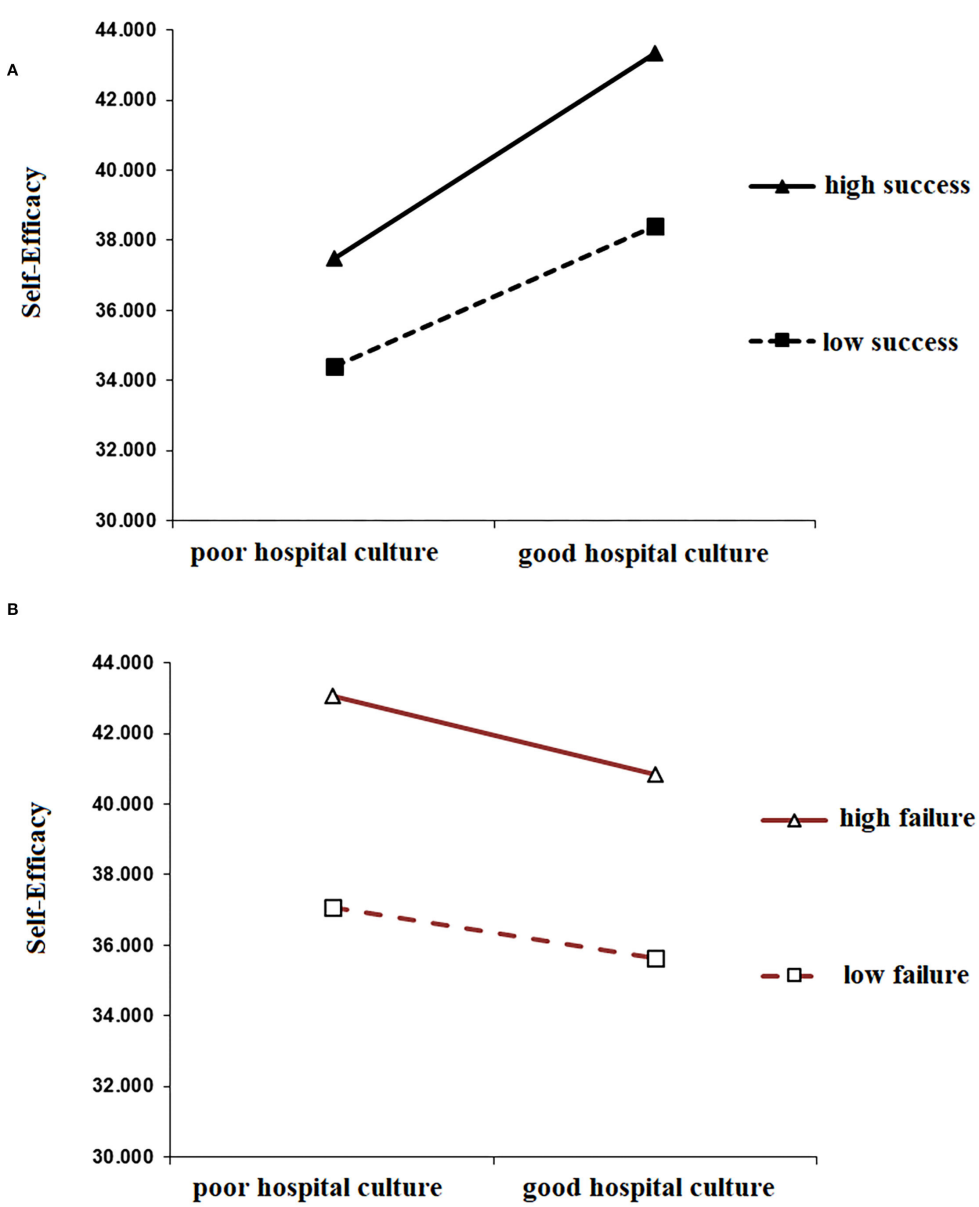
**(A)** Moderating effect of the pursuit of success in the relationship between hospital culture and healthcare workers' self-efficacy. **(B)** Moderating effect of the avoidance of failure in the relationship between hospital culture and healthcare workers' self-efficacy.

The moderating effect of healthcare workers' achievement motivation was examined using the mediation model. The two dimensions of achievement motivation were analyzed separately as the pursuit of success was positively correlated with the perceived provision of PCC, whereas the avoidance of failure was negatively correlated with the perceived provision of PCC. A moderated mediation model was constructed after decentering the pursuit of success, avoidance of failure, and hospital culture and including the interaction terms. The results of the moderated mediation model have been displayed in Table 6. The interactions between hospital culture and the pursuit of happiness and the avoidance of failure had significant effects on self-efficacy (β = 0.022, *p* < 0.001; β = −0.493, *p* < 0.001). Simple slope tests (Figure 2A) further revealed that hospital culture had a relatively weaker effect on healthcare workers' self-efficacy when the pursuit of success was low whereas hospital culture had a relatively stronger effect on healthcare workers' self-efficacy when the pursuit of success was high. Furthermore, Figure 2B shows that hospital culture had a relatively stronger effect on healthcare workers' self-efficacy when the avoidance of failure was low, whereas hospital culture had a relatively weaker effect on healthcare workers' self-efficacy when the avoidance of failure was high. Thus, these results supported H5, indicating that achievement motivation moderated the relationship between hospital culture and healthcare workers' self-efficacy. Additionally, the pursuit of success had a positive effect in the relationship between the aforementioned variables, whereas the avoidance of failure had a negative effect.

**Table 6:** Analysis of the moderated mediation effect.

**Antecedent**	**Consequent**					
	**M (self-efficacy)**			**Y (PCC)**		
	**β**	**SE**	***t***	**β**	**SE**	***t***
Constant	35.896	5.724	52.967^***^	49.26	1.425	34.567
Age	0.119	0.202	0.589	0.493	0.264	1.864
Title	0.74	0.178	4.148^***^	0.364	0.235	1.549
Post	−0.603	0.164	−3.664^***^	−0.747	0.217	−3.449^***^
Familiarity with PCC	0.62	0.137	4.510^***^	0.26	0.181	1.432
Hospital culture	0.405	0.017	24.047^***^	0.593	0.026	22.997^***^
Self-efficacy				0.403	0.031	12.938^***^
Pursuit of success	0.211	0.041	5.171^***^	1.064	0.252	4.217^***^
Avoidance of failure	−0.493	0.223	−2.209^***^	0.574	0.218	2.631**
Hospital culture × Pursuit of success	0.022	0.043	3.479^***^			
Hospital culture × Avoidance of failure	0.074	0.014	−7.979^***^			
Self-efficacy × Pursuit of success				0.128	0.115	4.385^***^
Self-efficacy × Avoidance of failure				−0.017	0.005	−3.327^***^
	R^2^ = 0.377			R^2^ = 0.522		
	F (7, 1722) = 149.017^***^			F (8, 1721) = 234.921^***^		

All values have been rounded up to three decimal places. ^*^p < 0.05, ^**^p < 0.01, and ^***^p < 0.001. Boot, bias-corrected percentile bootstrap method; SE, standard error; CI, 95% confidence interval; PCC, patient-centered care.

Moreover, Table 6 demonstrates that the interaction effects between self-efficacy and the pursuit of success and avoidance of failure had significant moderating effects on PCC (β = −0.128, *p* < 0.001; β = −0.017, *p* < 0.001). These results indicated that the mediating effect of self-efficacy in the relationship between hospital culture and the provision of PCC was moderated by achievement motivation (comprising the pursuit of success and avoidance of failure). To further evaluate the moderating effect of achievement motivation on the mediating effect of self-efficacy, we performed bootstrap testing and explored the indirect effect of the pursuit of success and avoidance of failure on three levels of self-efficacy. As shown in Table 7, the results demonstrate that self-efficacy had a stronger mediating effect on the perceived provision of PCC among healthcare workers with a high need for pursuing success (ρ = 0.340, 95% CI not including 0) as compared to healthcare workers with a low need for pursuing success (ρ = 0.190, bootstrap 95% CI not including 0). On the contrary, self-efficacy had the weakest mediating effect on the perceived provision of PCC among healthcare workers with a high need for avoidance of failure; that is, the indirect effect of self-efficacy was lowest when the avoidance of failure was 1SD above the mean (ρ = 0.153, 95% CI not including 0).

**Table 7:** Bootstrap testing for the moderated mediation effect.

**Moderating variable**	**Boot SE**	**Effect**	**Bootstrap 95% CI**	
			**Lower end of Boot CI**	**Higher end of Boot CI**
**Pursuit of success**				
−1SD (14.253)	0.036	0.190	0.12	0.259
M (16.899)	0.033	0.265	0.199	0.331
+1SD (19.546)	0.037	0.340	0.268	0.413
**Avoidance of failure**				
−1SD (6.637)	0.023	0.190	0.148	0.239
M (9.627)	0.02	0.172	0.135	0.215
+1SD (12.616)	0.028	0.153	0.105	0.217

*Boot, bias-corrected percentile bootstrap method; SE, standard error; CI, 95% confidence interval. All values have been rounded up to three decimal places*.

## Discussion

### Effects of Hospital Culture on PCC

The present study found that hospital culture played an important role in healthcare workers' care delivery. As a manifestation of healthcare personnel's collective values and norms, hospital culture can deeply influence their mindset and behaviors. Consistent with Zhang's study (13), innovative organizational culture was found to have a positive effect on healthcare workers' provision of PCC. This could be because an open atmosphere stimulates positivity and creativity among employees, creating a favorable environment for them to work on new projects and develop new skills, and especially to implement innovative patient-focused treatment plans. Additionally, the present findings are corroborated by those of Zhu's study (14) as charismatic leadership facilitated empathy for patients among healthcare workers; participants reported that they could think from the patients' perspective, and listen to and communicate effectively with them. Leaders effectively enhanced their employees' awareness of PCC and the importance of empathy by urging them to respect and care about patients and protect patients' rights.

Healthcare workers who had received training to develop their communication skills scored higher than those who had not received such training. This finding is consistent with Jeong and Park's study (49), suggesting that a healthy atmosphere involving communication is conducive to healthcare workers' efficiency in the provision of healthcare services as it boosts understanding and togetherness, fostering tolerance and unity. Additionally, in line with the conclusions drawn by Fralicx (50), cross-team collaboration was associated with effective communication among healthcare workers. Thus, hospitals can set up multidisciplinary teams and construct streamlined communication or feedback mechanisms to enhance cooperation among different departments and facilitate the implementation of PCC.

### Mediating Effects of Self-Efficacy

While exploring the mechanisms for the adoption of PCC, it is beneficial to examine the mediating effects of self-efficacy in the relationship between hospital culture and healthcare personnel's provision of PCC. Consistent with Yang et al. (51), the current study revealed that hospital culture affected healthcare workers' perceived provision of PCC, which was mediated by their self-efficacy. Self-efficacy can have direct effects on an individual's choices, goals, mindset, and attribution style, among others. Good hospital culture can foster healthcare professionals' self-efficacy, further enabling them to implement PCC.

The positive influence of hospital culture on self-efficacy can be explained in two ways. First, a supportive environment within a healthy hospital culture could increase healthcare workers' self-efficacy (52). When leaders highlight and champion the use of PCC in addition to advances in medical technologies and services that empower cross-team collaboration, healthcare workers might experience high levels of meaningfulness and confidence in completing their work; this could accelerate their ability to deliver healthcare services. Second, communication is an important factor in patient-centered medicine and calls for healthcare personnel's transition from traditional attitudes to attitudes of respect and care for patients' preferences, needs, and values (53). Open-minded leaders, innovative culture, and multidisciplinary cooperation can boost medical workers' confidence in effective communication, and thus, increase their willingness to implement PCC.

Meanwhile, self-efficacy had a positive effect on PCC; this is consistent with Sommaruga et al.'s findings (45), suggesting that healthcare providers with high self-efficacy had good interpersonal relationships and professional skills. Welsh (54) suggested that the enhancement of self-efficacy among doctors was effective in improving their communication skills; for example, doctors with high self-efficacy—consistent with the requirements of PCC—provided more disease-related information and medical knowledge to patients; encouraged them to communicate; and were responsive to their questions, suggestions, needs, and worries.

### Moderating Effects of Achievement Motivation

According to McClelland's (45) achievement need theory, achievement motivation among social members stems from specific social and cultural environments. In this study, we constructed a moderated mediation model based on achievement theories. Achievement motivation exerted significant moderating effects in the relationship between hospital culture and self-efficacy. In the same culture, healthcare workers with high achievement motivation and self-efficacy were highly willing to communicate with patients. This could be because they cared about the patients' feelings and were mindful about responding to them and meeting patients' preferences and needs. This is in line with Wang's study (20), suggesting that healthcare workers' self-efficacy, and hence, achievement motivation, are reinforced when they overcome problems and difficulties in services. On the contrary, when issues keep sustaining, achievement motivation is triggered among healthcare workers with high self-efficacy owing to their confidence, whereas healthcare workers with low self-efficacy might adopt negative attitudes concerning the provision of PCC to avoid failure.

In line with Khongsamai et al.'s findings (55), in the current study, an innovative organizational culture contributed to an increase in effective communication. This could be because an open and innovative work environment facilitates initiatives among healthcare workers, improves their consciousness about communication, fosters innovation in communication styles and skills, and thus, promotes doctor-patient communication. On the contrary, achievement motivation moderated the relationship between self-efficacy and patient-centered practice among healthcare workers. Similar to Yim and Lee's findings (56), we found that healthcare workers who pursued success tended to have strong social responsibility, introspected about their actions based on patients' feedback, showed progress in ethical values and skills, and encouraged patients to participate in discussions and express their feelings.

The avoidance of failure was found to negatively moderate the relationship between self-efficacy and the provision of PCC; this has repeatedly been demonstrated in the fields of psychology and education (57, 58). In the current study, this could be because medical personnel with a strong need to avoid failure might have anticipated job burnout owing to the patient-centered services that might require several emotional resources in addition to concentration; they might also have been worried about the status of rewards and outcomes of the long-term input. However, the negative effect of avoidance of failure was not stronger than the positive effect of pursuit of success, implying that the healthcare workers' need to pursue success primarily influenced their achievement motivation.

### Implications and Limitations

We believe that the present results have theoretical and practical significance. To begin with, the findings have strong implications for the development of strategies for stimulating healthcare workers' provision of PCC, which might be effective in transforming hospital administration methods and enhancing the efficiency of healthcare services. For example, to strengthen patient services, hospitals should establish an innovative culture, constantly work on creativity, encourage healthcare workers to innovate, and stimulate healthcare workers' internal achievement motivation by challenging them. Second, hospitals should establish a charismatic leadership culture. Hospital leaders should set an example and fully mobilize healthcare workers' consciousness and enthusiasm to serve patients. Moreover, hospital leaders should pay attention to their own quality improvement, respect healthcare workers, and be sensitive to their needs. Third, hospitals should establish a multidisciplinary diagnosis and treatment team to foster a culture of cooperation. By facilitating information exchange and providing a sharing platform for each department, a good communication and feedback mechanism is formed within the hospital, promoting internal cooperation. A good internal communication atmosphere is helpful for healthcare workers to understand each other, cultivate team spirit, and improve patient service efficiency. Our study found that the external hospital culture drives healthcare workers' attitudes and behaviors. It is not combined with the viewpoint of motivation theory, which adds further value to the literature on motivation theories.

The study also has some limitations. First, the recruited healthcare workers were from hospitals in Hangzhou; this could limit the representativeness of the sample and generalizability of our findings. Thus, further research should be conducted in other regions of China. Second, this study adopted a cross-sectional design; consequently, we could not capture the causal effect of changes over time. Therefore, future studies must adopt longitudinal, experimental, or cross-sequential designs and employ hierarchical linear models; studies must also test for the confounding and mediating effects of different variables. Third, this study explored healthcare workers' perceptions of their provision of PCC; future studies must focus on the perspectives of both healthcare providers and patients. Fourth, all the variables were assessed through self-report, and although surveys were answered anonymously, social desirability bias may still have influenced the responses to some extent.

## Conclusions

The present study indicated that hospital culture can affect healthcare workers' implementation of PCC. Accordingly, hospitals could organize activities for healthcare workers to discuss hospital culture; this would strengthen their understanding of the importance of hospital culture. Additionally, hospital culture can boost the provision of PCC *via* the enhancement of self-efficacy and achievement motivation among healthcare workers. Therefore, hospital administrators should pay attention to the psychological status of their staff and develop their confidence in interpersonal networking and effective communication to help them build their self-efficacy for PCC and motivation for success.

## Data Availability Statement

The original contributions presented in the study are included in the article/supplementary material, further inquiries can be directed to the corresponding author/s.

## Ethics Statement

The study was approved by the Institutional Review Board of Hangzhou Normal University. All study participants provided informed consent, and the study was performed in accordance with the Ethical Standards as laid down in the 1964 Declaration of Helsinki and its later amendments. The patients/participants provided their written informed consent to participate in this study.

## Author Contributions

XH conceptualized the study, drafted the methodology, and operated the software. YG performed the statistical analysis and prepared the first draft of the manuscript. HC helped with visualization and investigation. HZ operated the software and validated the findings. XZ helped in writing, reviewing, and editing the manuscript. All authors approved the submitted version of the manuscript.

## Funding

This study was supported by the National Natural Science Foundation of China Project (Grant No. 72004051) and the Soft Science Research Program of Zhejiang Provincial Science and Technology Plan (Grant No. 2021C35012).

## Conflict of Interest

The authors declare that the research was conducted in the absence of any commercial or financial relationships that could be construed as a potential conflict of interest. The reviewer XW declared a shared affiliation with all authors at the time of review.

## Publisher's Note

All claims expressed in this article are solely those of the authors and do not necessarily represent those of their affiliated organizations, or those of the publisher, the editors and the reviewers. Any product that may be evaluated in this article, or claim that may be made by its manufacturer, is not guaranteed or endorsed by the publisher.
